# Supplementary material for: Potent Anti-Cancer Activity of 1-Dehydrodiosgenone from the Product of Microbial Transformation of Steroid Saponins
Source: Int J Mol Sci. 2024 Dec 6;25(23):13118. doi: 10.3390/ijms252313118 (PMC11641788; doi:10.3390/ijms252313118)
Supplement: Supplementary file 1 [file ijms-25-13118-s001.zip › ijms-3330904-supplementary.pdf]

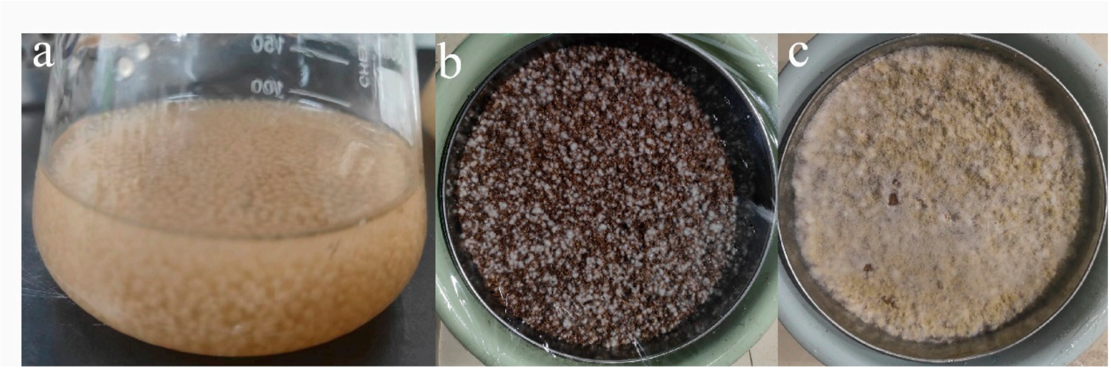

Figure S1. solid-state fermentation of strain SY$_{fxl}$23.3; (a) SY$_{fxl}$23.3 secondary seed liquid; (b) SY$_{fxl}$23.3 strain for solid-state fermentation for 2 days; (c) SY$_{fxl}$23.3 strain for solid-state fermentation for 7 days

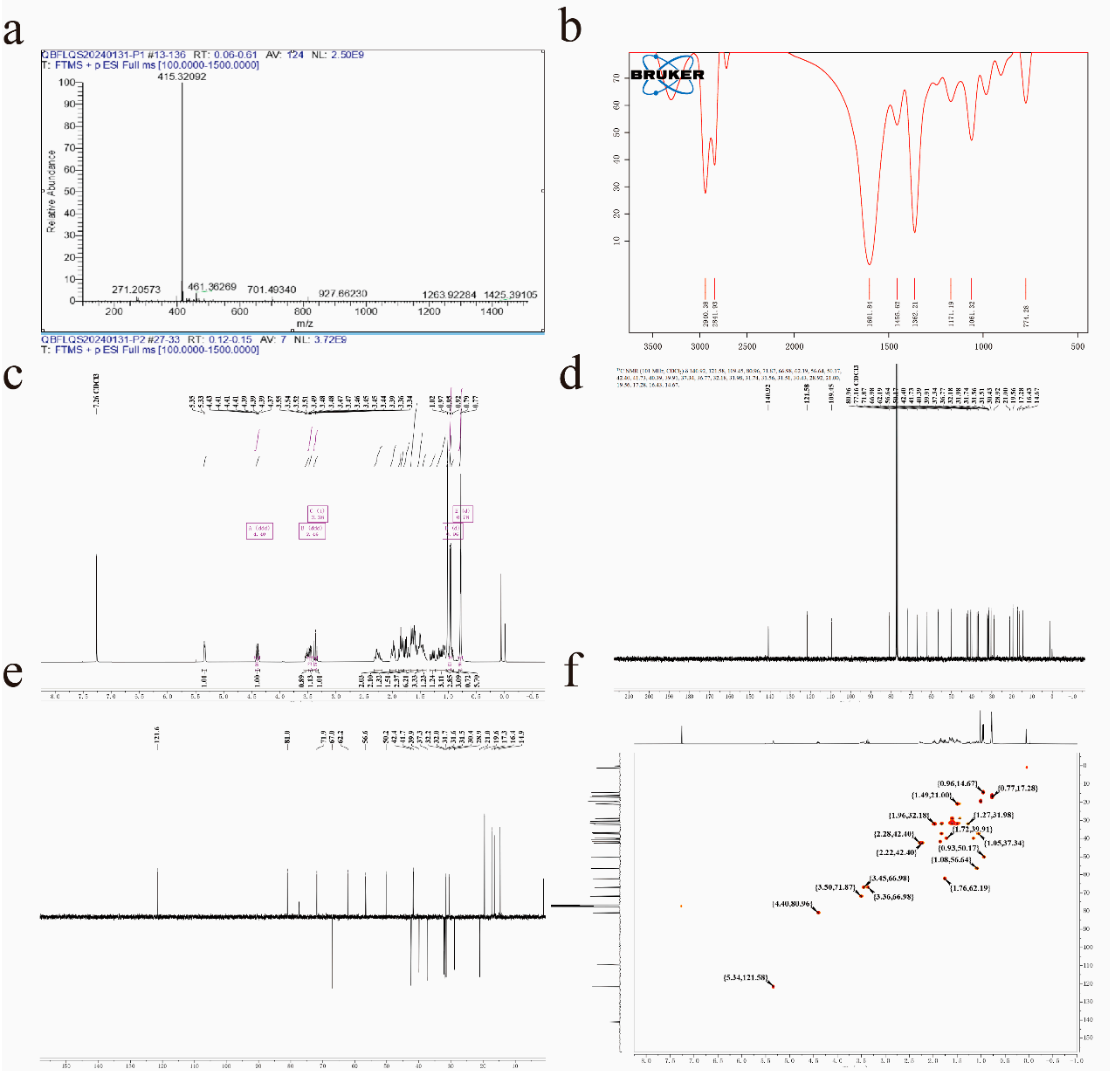

Figure S2. Group of spectra for diosgenin detection. (a) LC-MS spectrum of diosgenin; (b) Fourier-transform infrared (FT-IR) spectrum of diosgenin; (c) ^{1}H-NMR spectrum of diosgenin; (d) ^{13}C NMR spectrum of diosgenin; (e) DEPT135° spectrum of diosgenin; (f) HSQC 2D spectrum of diosgenin

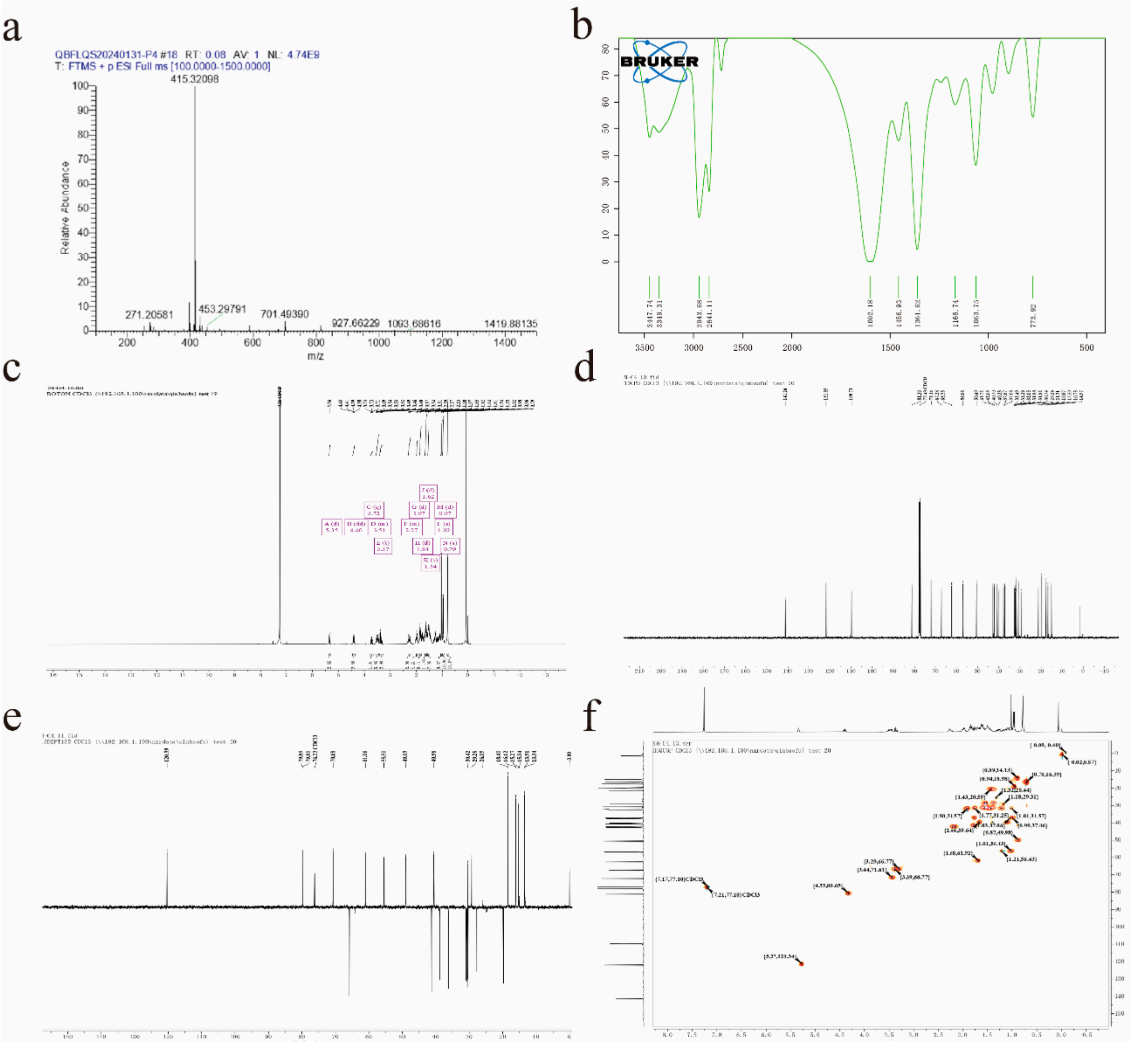

Figure S3. Group of spectra for Yamogenin detection. (a) LC-MS spectrum of Yamogenin; (b) Fourier-transform infrared (FT-IR) spectrum of Yamogenin; (c) ^{1}H-NMR spectrum of Yamogenin; (d) ^{13}C NMR spectrum of Yamogenin; (e) DEPT135° spectrum of Yamogenin; (f) HSQC 2D spectrum of Yamogenin

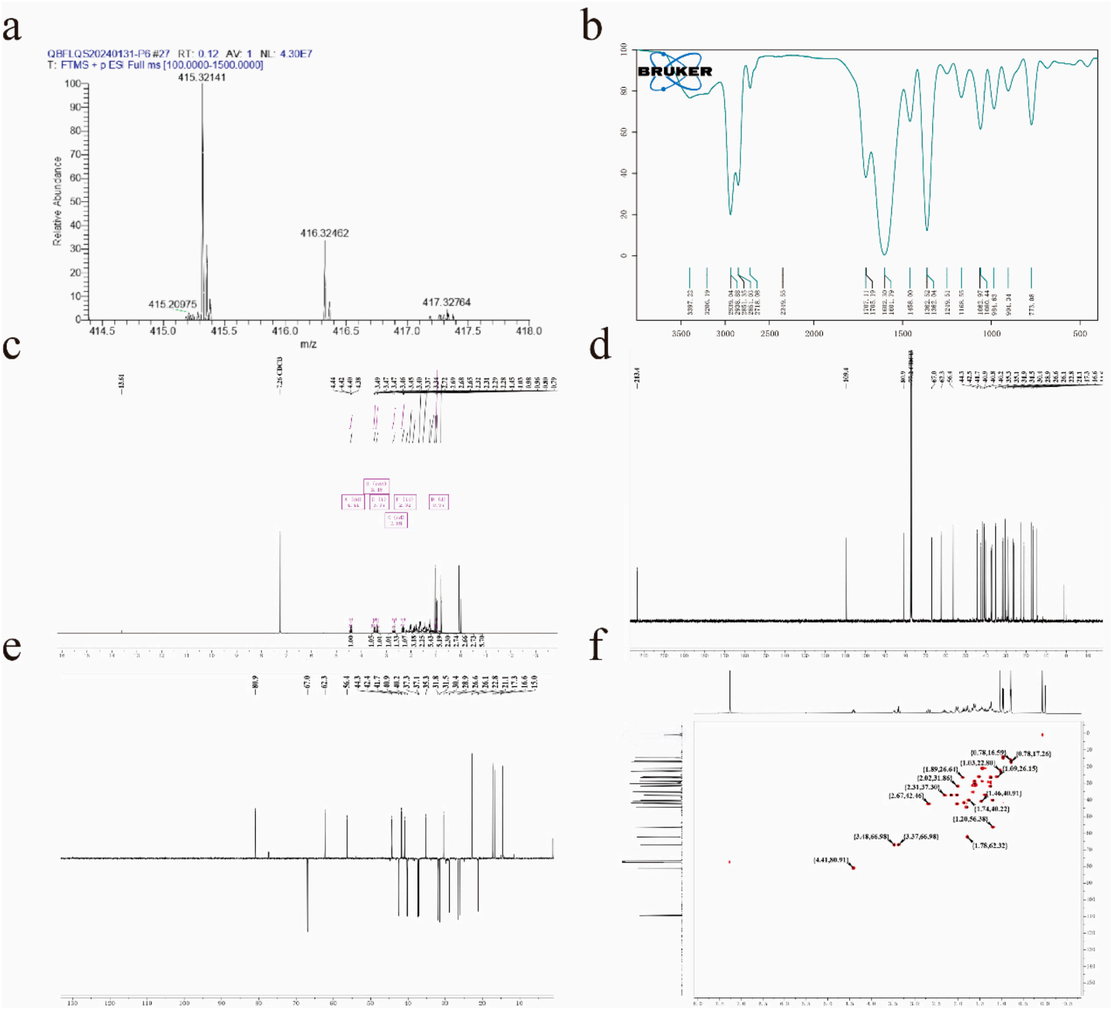

Figure S4. Group of spectra for Smilagenone detection. (a) LC-MS spectrum of Smilagenone; (b) Fourier-transform infrared (FT-IR) spectrum of Smilagenone; (c) ^{1}H-NMR spectrum of Smilagenone; (d) ^{13}C NMR spectrum of Smilagenone; (e) DEPT135° spectrum of Smilagenone; (f) HSQC 2D spectrum of Smilagenone

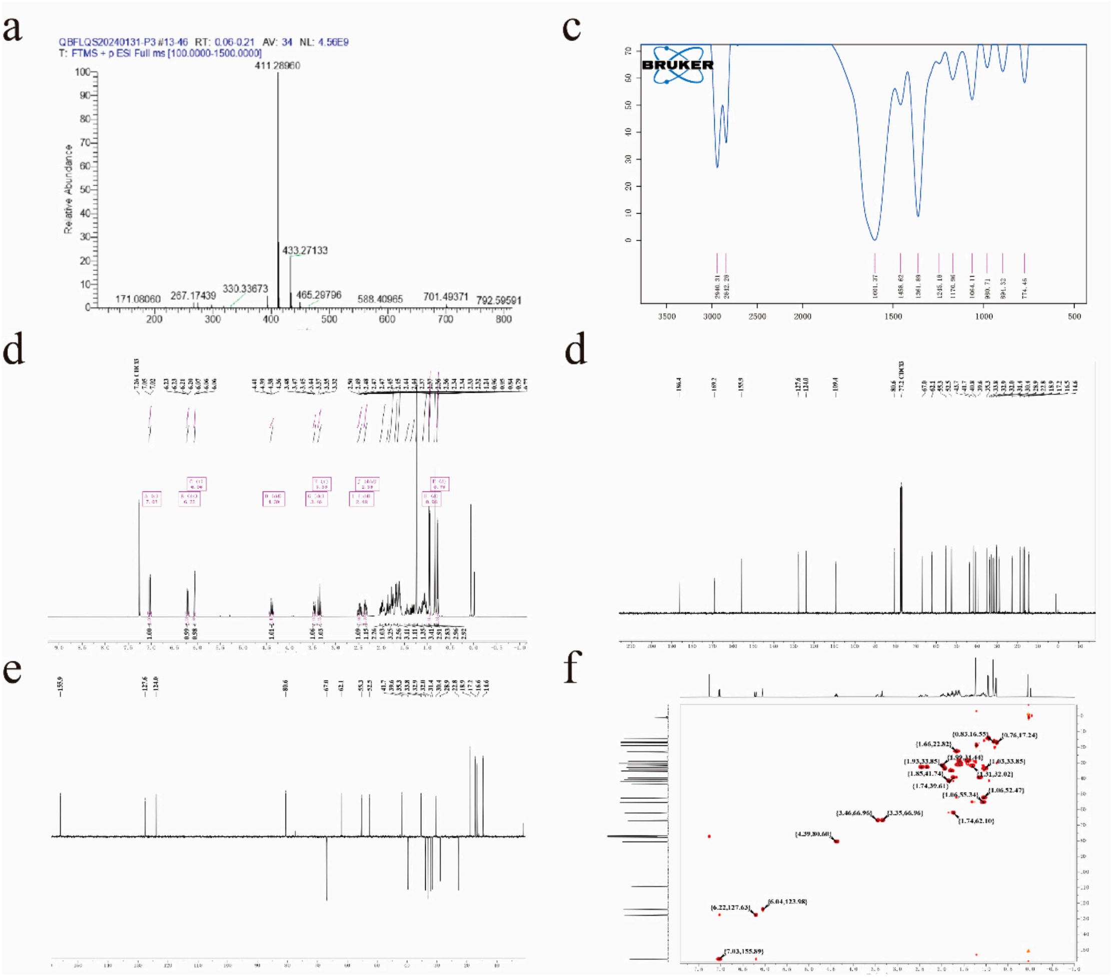

Figure S5. Group of spectra for 1-Dehydrodiosgenone detection. (a) LC-MS spectrum of 1-Dehydrodiosgenone; (b) Fourier-transform infrared (FT-IR) spectrum of 1-Dehydrodiosgenone; (c) ^{1}H-NMR spectrum of 1-Dehydrodiosgenone; (d) ^{13}C NMR spectrum of 1-Dehydrodiosgenone; (e) DEPT135° spectrum of 1-Dehydrodiosgenone; (f) HSQC 2D spectrum of 1-Dehydrodiosgenone

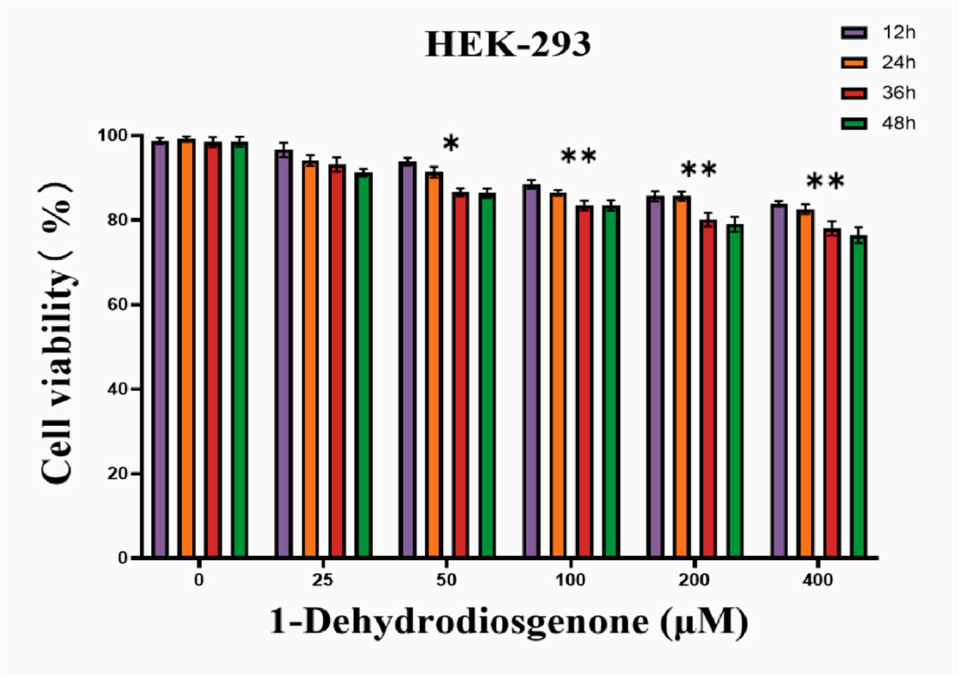

Figure S6. Cytotoxic activity of 1-dehydrodiosgenone against HEK-293 (human embryonic kidney cells) in vitro.

Table S1. Crystal data and structure refinement of compound **3**

CCDC number	CCDC-2340543
Empirical formula	$C_{27}H_{42}O_3$
Formula weight	414.60
Temperature [K]	100.00
Crystal system	orthorhombic
Space group (number)	$P2_12_12_1$
a [Å]	7.2125(14)
b [Å]	14.343(2)
c [Å]	22.527(4)
α [°]	90
β [°]	90
γ [°]	90
Volume [$Å^3$]	2330.4(7)
Z	4
ρ_{calc} [gcm^{-3}]	1.182
μ [mm^{-1}]	0.578
F(000)	912
Crystal size [mm^3]	0.16×0.15×0.13
Radiation	CuK_α (λ=1.54178 Å)
2θ range [°]	7.31 to 136.46 (0.83 Å)
Reflections collected	65578
Independent reflections	4279，R_{int} = 0.1126，R_{sigma} = 0.0512
Data / Restraints / Parameters	4279/0/276
Goodness-of-fit on F^2	1.122
Final R indexes [I≥2σ(I)]	R_1 = 0.0621，wR_2 = 0.1308
Final R indexes [all data]	R_1 = 0.0818，wR_2 = 0.1492

Table S2. Crystal data and structure refinement of compound **4**

CCDC number	CCDC-2325701
Empirical formula	$C_{27}H_{38}O_3$
Formula weight	410.57
Temperature/K	100.00
Crystal system	orthorhombic
Space group	$P2_12_12_1$
a/Å	7.2305(3)
b/Å	14.7863(7)
c/Å	21.3686(7)
α/°	90
β/°	90
γ/°	90
Volume/Å^3	2284.57(16)
Z	4
ρ_{calc}g/cm^3	1.194
μ/mm^{-1}	0.589
F(000)	896.0
Crystal size/mm^3	0.18 × 0.16 × 0.14
Radiation	Cu Kα (λ = 1.54178)
2Θ range for data collection/°	7.27 to 148.936
Reflections collected	120194
Independent reflections	4658 [R_{int} = 0.0381, R_{sigma} = 0.0302]
Data/restraints/parameters	4658/0/276
Goodness-of-fit on F^2	1.114
Final R indexes [I>=2σ (I)]	R_1 = 0.0313, wR_2 = 0.0751
Final R indexes [all data]	R_1 = 0.0313, wR_2 = 0.0751
